# Double–Multiplex Immunostainings for Immune Profiling of Invasive Breast Carcinoma: Emerging Novel Immune-Based Biomarkers

**DOI:** 10.3390/ijms26072838

**Published:** 2025-03-21

**Authors:** Sofia D. P. Theodorou, Konstantinos Ntostoglou, Ilias P. Nikas, Dimitrios Goutas, Vassilis Georgoulias, Christos Kittas, Ioannis S. Pateras

**Affiliations:** 1 Medical School, National and Kapodistrian University of Athens, 11527 Athens, Greece; sofiatheo8@gmail.com (S.D.P.T.); kostasntostoglou@gmail.com (K.N.); ckittas@med.uoa.gr (C.K.); 2 Medical School, University of Cyprus, 2029 Nicosia, Cyprus; nikas.ilias@ucy.ac.cy; 3 2nd Department of Pathology, “Attikon” University Hospital, Medical School, National and Kapodistrian University of Athens, 12462 Athens, Greece; goutas.dimitris@hotmail.com; 4 Hellenic Oncology Research Group (HORG), 11474 Athens, Greece; georgulv@otenet.gr

**Keywords:** double immunostaining, multiplex immunostaining, invasive breast carcinoma, T lymphocytes, macrophages, biomarker, digital pathology, artificial intelligence, precision therapy

## Abstract

The role of tumor microenvironment in invasive breast cancer prognosis and treatment is highly appreciated. With the advent of immunotherapy, immunophenotypic characterization in primary tumors is gaining attention as it can improve patient stratification. Here, we discuss the benefits of spatial analysis employing double and multiplex immunostaining, allowing the simultaneous detection of more than one protein on the same tissue section, which in turn helps us provide functional insight into infiltrating immune cells within tumors. We focus on studies demonstrating the prognostic and predictive impact of distinct tumor-infiltrating lymphocyte subpopulations including different CD8(+) T subsets as well as CD4(+) T cells and tumor-associated macrophages in invasive breast carcinoma. The clinical value of immune cell topography is also appreciated. We further refer to how the integration of digital pathology and artificial intelligence in routine practice could enhance the accuracy of multiplex immunostainings evaluation within the tumor microenvironment, maximizing our perception of host immune response, improving in turn decision-making towards more precise immune-associated therapies.

## 1. Introduction

Invasive breast carcinoma is the second cause of cancer incidence worldwide in 2022, comprising 11.6% of all cancer diagnoses [1]. Breast cancer is the most commonly diagnosed malignancy in women, accounting for almost one in four cancer cases, and it is the leading cause of cancer death among women [1]. Notably, by 2060 in low- and low–middle-income countries, breast cancer is projected to be the single greatest contributor to serious health-associated issues among cancer patients, thereby stressing the necessity of improving therapeutic strategies [2]. In addition, with aging, breast cancer incidence is rising, with the estrogen-positive breast cancer subtypes rising during post-menopausal years [3].

Tumor node metastasis, lymphovascular invasion, and grading, along with estrogen receptor (ER) and progesterone receptor (PR), human epidermal growth factor receptor 2 (HER2), and the proliferation index (Ki-67), are established prognostic and predictive markers employed in routine practice for the stratification of breast cancer patients [4,5,6,7]. Accumulating evidence supports the prognostic and predictive role of tumor microenvironment (TME) in invasive breast carcinoma, although this knowledge has not yet been translated into clinical practice [8,9,10]. Specifically, the density of tumor-infiltrating lymphocytes (TILs) is gaining attraction as a prognostic and predictive factor in breast cancer. High TIL infiltration is associated with a favorable outcome and better response to neoadjuvant chemotherapy in triple-negative and HER2-positive breast cancer patients [9]. The latter suggests the importance of understanding TME to develop novel therapeutic strategies that could modulate immune response.

Spatial analysis helps us understand the complex interactions between cancer cells with the surrounding tissue, including infiltrating immune cells. In addition, an analysis of intact tissues helps us capture TME heterogeneity. In this context, single immunostainings, including immunohistochemistry (IHC) and immunofluorescence (IF) employing chromogenic or fluorescent dyes, respectively, permits the analysis of one antigen per section. To improve our perception of TME, the employment of double–multiplex immunostainings offers the ability to simultaneously detect more than one protein biomarker on the same tissue section, which in turn allows us to dissect the immune landscape with single-cell resolution [11]. Moreover, computational approaches enable the geographical conceptualization of the TME of tissue sections that may not be feasible during manual microscopy [12]. Along this line, the integration of digital pathology and artificial intelligence (AI) enhances the evaluation of double and multiplex IHC or IF, advancing our understanding of the tumor microenvironment and the discovery of novel prognostic and therapeutic biomarkers in various cancers [12].

Excellent reviews describing multiplex staining techniques can be found elsewhere [11,13]. This manuscript focuses on emerging T cell- and macrophage-associated biomarkers based on double–multiplex immunostaining approaches that could improve clinical stratification in invasive breast carcinoma. Furthermore, improvements gained by digital pathology and AI are appreciated.

## 2. Single Immunostaining: Assessing One Dimension

Conventional IHC and IF have been extensively employed to study TME in invasive breast carcinoma. As CD8(+) T lymphocytes are key effector immune cells, most studies have assessed the clinical impact of CD8(+) cells in different breast cancer subtypes. Following early observations in sporadic colorectal cancer [14,15], increased infiltration by CD8(+) T cells is associated with a favorable clinical outcome in breast cancer [16]. Even though CD8(+) status is a surrogate marker of anti-tumor immunity, increased intratumoral infiltration of CD8(+) T lymphocytes does not necessarily mean that this population is functional [17]. It has been recognized that CD8(+) T cells within TME are associated with an altered functional state. A fraction of dysfunctional T cells have been observed in different tumors including breast cancer tumors [17]. T cell exhaustion represents a dysfunctional T cell state that has been demonstrated in breast cancer patients [18]. To define T cell exhaustion, more than one marker needs to be assessed simultaneously on the same tissue section, stressing the necessity of double–multiplex immunostainings. Moreover, to study different immune subpopulations and assess expression patterns and interaction between various immune cells, the labeling of multiple markers on a single tissue section is required.

## 3. Principles of Double–Multiplex Immunostainings

The employment of double–multiplex immunostaining allows us to dissect the immune landscape in TME through the simultaneous detection of two or more markers on individual cells in the same section [11]. In principle, double–multiplex immunohistochemistry (IHC) and immunofluorescence (IF) techniques employ advanced stain removal and chromophore/fluorophore inactivation technologies to facilitate the simultaneous detection of multiple biomarkers within a single tissue section. Stain removal technologies enable sequential staining, imaging, and stain elimination, allowing for the iterative detection of various markers [11]. Chromogenic IHC is suitable for the simultaneous detection of 2–3 protein markers, while fluorescence platforms offer high-multiplex options [11]. Cumulatively, multiplex immunostaining has several advantages: (a) it allows the phenotypic characterization of different immune subtypes, providing functional insight; (b) since the analysis is performed in intact tissues, the assessment of tumor–immune interaction is feasible in providing insight into immune cell topography; and (c) it preserves tissue in small biopsies during routine practice. Furthermore, with the advent of immunotherapy, it is important to develop a panel of immune-related markers with prognostic and predictive impacts that improve treatment decisions.

## 4. Clinical Value of Double–Multiplex Immunostainings in Invasive Breast Carcinoma

In 2018, Grigor et al. developed a multiplex panel, consisting of Programmed Death-1 (PD-1), Programmed Death Ligand 1 (PD-L1), OX40, CD27, T-cell immunoglobulin, and mucin domain-containing-3 (TIM-3), along with the T cell marker CD3, allowing the detection of several immune checkpoint molecules in different cancers including invasive breast ductal carcinoma [11]. The authors stained formalin-fixed paraffin-embedded tissues (FFPE), employing tyramide signal amplification that allows the detection of low-abundantly expressed proteins. Below, we provide an update on manuscripts published on PubMed focusing on distinct subpopulations of T lymphocytes infiltrating tumors and macrophages with prognostic and predictive impact in formalin-fixed paraffin-embedded (FFPE) tissues from patients with invasive breast carcinoma (Table 1).

The role of FOXP3(+) regulatory cells in breast carcinoma has been largely appreciated. A high CD8(+)/FOXP3(+) ratio and a high CD8(+) T cell count are both associated with favorable recurrence-free survival (RFS) and breast cancer-specific survival (BCSS) in a cohort of triple-negative breast cancer (TNBC) patients that received neoadjuvant chemotherapy (the majority received the standard regimen including anthracyclines and taxanes) [19]. Hayashi and co-authors demonstrated that low CD4(+)FOXP3(+), CD8(+)FOXP3(+), CD4(+)PD-1(+), CD8(+)PD-1(+), and CD8(+)PD-L1(+) levels in TME in patients with TNBC are associated with a higher recurrence rate [20]. Interestingly, CD8(+)FOXP3(+) T cells share developmental similarities with CD4(+)FOXP3(+) T cells, although studies in mice show that they lack potent suppressant activities [33]. Their role in human TME is understudied [34]. Another study demonstrated that the dense infiltration of FOXP3(+) T cells co-expressing the IL-2 receptor α chain (CD25) in the primary tissue of patients with TNBC who received standard neoadjuvant chemotherapy is associated with favorable overall survival (OS) [21]. Notably, FOXP3+ alone did not have any impact on patient survival, highlighting the heterogeneity of regulatory T cells and the importance of using double–multiplex staining to understand the role of this T cell subtype in cancer. In the same study, the authors demonstrated that high PD-L1 expression, assessed separately in cancer cells and tumor-infiltrating lymphocytes (TILs), is associated with pathologic complete response (pCR) and improved OS [35]. Interestingly, a study based on 10,090 women from the Dutch population assessed whether TME could predict the transition from in situ to invasive breast carcinoma [36]. The authors employed multiplex immunostaining, including CD20(+) B, CD3(+)CD8(+) T, CD3(+)FOXP3(+) regulatory T cells, CD68(+) cells, and CD8(+)Ki67(+) proliferating T cells, and found that the assessment of these markers in the TME was not a predictor of invasive breast carcinoma development [36].

The assessment of different T cell states is challenging in TME. An analysis of CD8(+)PD-1(+) status in an Asian cohort of TNBC patients demonstrated that an increased density of CD8(+)PD-1(+), but not increased CD8(−)PD-1(+) infiltration, was associated with improved disease-free survival (DFS) [22]. PD-1 expression is associated with T cell exhaustion, and, therefore, one could expect that increased PD-1 co-expression by CD8(+) T lymphocytes would promote immune evasion. However, this is an oversimplification, as T cell exhaustion is a progressive and not a binary state [17]. Accumulating data distinguishes T cell exhaustion between early exhaustion (predysfunctional) and late (dysfunctional) exhaustion states [17]. Early-exhausted CD8(+) T cells are characterized by absent or decreased inhibitory receptor expressions, including PD-1 and TIM3, and self-renewal capacity demonstrated by T cell factor 1 (TCF1) expression. On the other hand, late-exhausted T cells exhibit increased inhibitory expression and have a decreased proliferative potential [37]. Therefore, PD-1 expression may reflect an early or late exhaustion state, providing an explanation for the favorable prognostic impact of CD8(+)PD-1(+) status. In addition, it has been shown that PD-1 expression can identify clonally expanded tumor-reactive T cells in melanoma patients, showing the complexity of PD-1’s role in TME [38]. Along this line, the amount of CD4(+)PD-1(+) and CD8(+)PD-1(+) is higher in TNBC versus luminal type A breast cancer patients, showing that in TNBC patients, the PD-1/PD-L1 axis is a target for immune checkpoint inhibitors [39]. Notably, PD-1(+), CD8(+)PD-1(+), and CD8(+)PD-1(+)/CD8(+) status has a predictive value; PD-1(+), CD8(+)PD-1(+) density, and the CD8(+)PD-1(+)/CD8(+) ratio is higher in pre-neoadjuvant-treated tumors from responders versus non-responders, while CD8(+)TIM3(+) status failed to predict response to neoadjuvant therapy [23]. We recently demonstrated that the increased density of CD8(+)TCF1(+) T cells in the tumor center is an independent prognostic biomarker linked with improved DFS only in TNBC but not in luminal type A breast cancer patients [24], showing the importance of providing a functional insight into CD8(+) cells rather than merely counting their numbers. In this context, Wang et al. demonstrated that an increased density of proliferating [Ki-67(+)] CD8(+)TCF1(+) T cells is a favorable predictor of response to immunotherapy in TNBC [31]. Their cohort included 243 TNBC patients enrolled in the NeoTRIP randomized controlled trial, which compared neoadjuvant chemotherapy (i.e., carboplatin and nab-paclitaxel) with chemotherapy along with anti-PD-L1 immunotherapy (i.e., atezolizumab) [30]. Notably, the authors showed that proliferating CD8(+)TCF1(+) T cells were often in contact with cancer cells expressing major histocompatibility complex II, thereby stressing the importance of evaluating cancer–immune interactions [31].

Resident memory T cells are another subpopulation of T cells that are associated with improved clinical outcomes in breast cancer [40]. T cell resident memory cells express high levels of immune checkpoint molecules (like PD-1, CTLA-4, and TIM-3), effector proteins (including granzyme B, perforin 1, and interferon γ), and CD69 and CD103 [40]. CD103 binds to E-Cadherin, which is expressed by epithelial cells, promoting the retention of these cells in the peripheral tissues [40]. Single-cell RNA profiling revealed that a high CD8(+) resident memory signature is associated with improved prognosis in early-stage TNBC patients [41]. This is in line with a previous study including 424 basal-like subtype breast cancer cases showing that increased intraepithelial CD103(+) T cells but not intrastromal T cells (employing single immunostaining) predicts better relapse-free survival and OS [42]. Future studies implementing double–multiplex immunostainings in intact tissues that will consider immune cell topography are expected to further shed light on the role of resident memory T cells in breast cancer.

Tumor-associated macrophages (TAMs) constitute a dominant population of immune cells within the TME [43]. TAMs share features of alternatively activated macrophages (also known as M2) macrophages [44]. M2 macrophages are involved in tissue repair and have anti-inflammatory and angiogenetic properties; therefore, they are considered pro-tumorigenic [44]. On the other hand, classically activated (also known as M1) macrophages are pro-inflammatory and favor tumor destruction [44]. As TAMs display an M2-like immunophenotype, they can be detected in the TME employing two common M2 markers, namely anti-CD206 and anti-CD163, which recognize mannose and hemoglobin/haptoglobin receptors, respectively [45]. Additional antibodies can be incorporated into the immunostaining approach of TAMs, including CD68(PGM1) [45]. Along this line, we recently depicted that an increased count of CD163(+) macrophages in the tumor center is associated with dismal prognosis only in luminal type A but not in TNBC patients [24]. This is in line with a recent meta-analysis showing that high-CD163(+) TAMs are associated with poor patient clinical outcomes in several tumors including breast cancer [46]. Moreover, prompted by previous studies depicting the co-expression of the immune checkpoint inhibitors PD-L1 or PD-1 by TAMs [47,48,49], we assessed the clinical impact of CD163(+)PD-L1(+) TAMs and found that increased infiltration by CD163(+)PD-L1(+) macrophages is related with a lower relapse rate in TNBC patients [24]. This latter point is in line with a study by Wang and co-authors showing that high stromal CD68(+)PD-L1(+) TAMs were associated with improved OS in TNBC [29]. Another study showed that the increased density of proliferating TAMs characterized by the presence of CD68(+) macrophages co-expressing the proliferating marker Proliferating Cell Nuclear Antigen (PCNA) is associated with an increased risk of death [25]. Along this line, high infiltration by CD68(+)PCNA(+) macrophages is associated with decreased recurrence-free survival (RFS) in patients with invasive breast carcinoma who received neoadjuvant chemotherapy [26]. Surprisingly, in this study, CD68(+)PCNA(+) TAMs were more associated with the M1-like phenotype rather than the M2-like phenotype, which is pro-tumorigenic [26]. Another study assessed the expression of the transmembrane protein CD47 by TAMs [27]. CD47 binds to signal-regulatory protein-alpha (SIRPα), triggering the “do not eat me signal” to macrophages that in turn inhibits phagocytosis [50]. High-CD68(+)CD47(+) TAMs were an independent prognostic factor associated with shorter RFS among all breast cancers as well as among luminal type A invasive breast carcinomas [27]. Finally, Esbona et al. demonstrated that the high infiltration of CD68(+)COX-2(+) in the tumor stroma and a high density of CD163(+)COX-2(+) TAMs in the tumor nests are associated with worse patient overall survival [28], which is in line with the previous studies regarding the prognostic role of TAMs.

Bady and co-authors developed a BLEACH&STAIN multiplex IF platform allowing the simultaneous detection of 15 immune-associated markers in the TME, incorporating AI-based frameworks with clinical impact [32]. Specifically, the authors assessed the status of PD-L1, PD-1, CTLA-4, CD68, CD163, CD11c, iNOS, CD3, CD8, CD4, FOXP3, CD20, and Ki67 along with pan Cytokeratin and CD31 in the TME of 3098 tumors, including 587 cases with breast cancer. In breast cancer, they demonstrated that inflamed tumors (i.e., having high CD8, CD4, FOXP3, and dendritic cell density) along with increased PD-L1 expression on tumor cells and immune cells were associated with prolonged OS [32].

Overall, these studies demonstrate the clinical value of implementing double–multiplex immunostaining, as it allows the characterization of distinct subpopulations of T cells and TAMs with prognostic and predictive impacts in invasive breast cancer. Importantly, it seems that the clinical impact of the immunophenotypic profile is different among different breast cancer subtypes. Additionally, accumulating evidence supports the clinical value of immune cell topography. Along this line, König et al. demonstrated the distinct distribution of TILs and TAMs among the tumor center and invasive front in 87 patients with invasive breast carcinoma who received neoadjuvant chemotherapy [51]. Specifically, the authors examined the status of CD3(+), CD4(+), CD8(+), CD20(+), and CD68(+) cells separately in the tumor center and the invasive front and demonstrated that increased CD3(+) T cell density at the tumor center is associated with a favorable response to neoadjuvant chemotherapy [51]. Another study depicted the prognostic impact of studying CD8(+), FOXP3(+), and CD163(+) cell density separately in the tumor center and the invasive front [52], stressing the significance of considering immune cell distribution.

## 5. Future Perspectives—Implementing Digital Pathology and Artificial Intelligence

Pathology has entered a new era with the emergence of digital pathology and AI. The application of digital pathology and AI implementation can offer pathologists and clinicians valuable information regarding the diagnosis, prognosis, and therapy of several malignancies [53,54].

**Figure 1:**
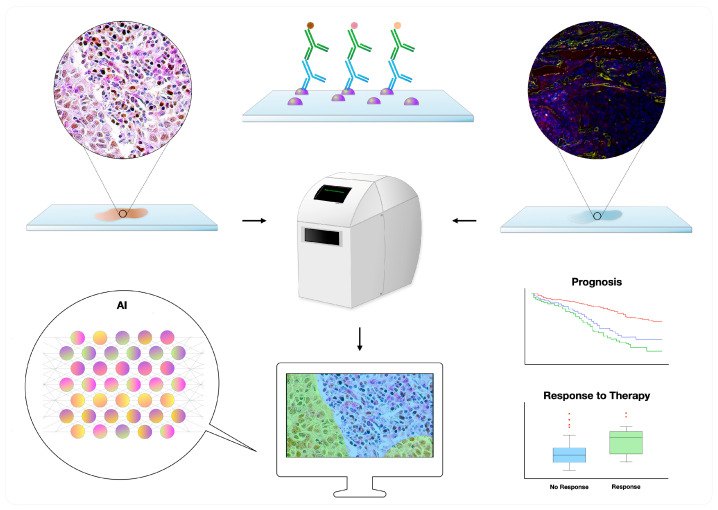
Digital pathology workflow of double–multiplex immunohistochemistry and immunofluorescence slides: The slides are first scanned and subsequently analyzed with the help of image analysis and artificial intelligence (AI), allowing the development of prognostic and predictive biomarkers. Multiplex immunofluorescence photo courtesy of Jean Descarpentrie and Teresa Frisan (Umeå University, Umeå, Sweden).

Glass slides stained with hematoxylin and eosin (H&E) or other techniques (e.g., IHC and IF) are first scanned, generating whole-slide images; the latter can then be evaluated with the aid of image analysis and AI (Figure 1) [12,53]. Several platforms exist in the market, supporting distinct diagnostic and research applications [12]. Of interest, a few AI algorithms have been approved by regulatory bodies such as the Food and Drug Administration (FDA) for diagnostic use, including the “Paige Prostate” for prostatic biopsies [55,56], analyzing H&E slides, and the “Genius Digital Diagnostics System” for cervical cytopathology, analyzing Papanicolaou-stained slides [57]. Other algorithms can augment selected tasks such as counting mitoses [58] and spotting metastatic deposits in patients with breast cancer [58], also predicting the presence of mutations (e.g., EGFR) by processing H&E slides [59]. Regarding IHC, selected algorithms can objectively detect and quantify immunomarkers, including ER, PR, HER2, and Ki-67 [59]. Apart from simply quantifying, it is also possible to assess a biomarker’s intratumoral heterogeneity, such as with Ki-67, which might be of prognostic significance in several scenarios [60,61].

There is a growing body of data demonstrating the implementation of digital pathology and AI to assess immune-based markers in the TME. To tailor patients for immunotherapy, these are often screened for the presence of various biomarkers, including PD-L1, tumor mutational burden (TMB), and mismatch repair deficiency/high levels of microsatellite instability (dMMR/MSI-H) [62]. Several AI algorithms quantifying the expression of PD-L1 exist in the market, allowing the application of methodologies such as combined positive score (CPS), Tumor Proportion Score (TPS), and others; for instance, the “uPath PD-L1 (SP263) image analysis tool” by Roche^®^ is a CE-IVD-certified tool for non-small-cell lung cancer [63,64]. Other algorithms could predict the presence of dMMR/MSI-H [65] or objectively quantify the TILs [65] or tertiary lymphoid structures [65]. In a research setting, digital pathology and AI have often been utilized by various groups to investigate the TME, including the expression of selected biomarkers (e.g., CD8 and Ki-67) in the tumor center besides its edge and interface zone, allowing the assessment of tissue topography [65,66,67,68]. Of interest, the spatial distribution of such biomarkers may carry prognostic significance [66,69].

Using AI algorithms to promote multiplex IHC or IF evaluation could revolutionize our understanding of the TME and facilitate the discovery of important spatial relationships and complex signaling pathways, becoming an important element of future diagnostic practice [12]. Potential clinical applications may include saving material from tissue biopsies for subsequent high-throughput molecular testing—through evaluating multiple markers in one tissue section rather than each marker in a separate section—or assessing multiple immunomarkers to predict response to immune checkpoint inhibitors [11]. For instance, multiplex IF panels composed of the PD-1, PD-L1, CD8, FOXP3, CD163 (or CD68), and Sox10/S100 (or Cytokeratin) markers have already been evaluated in large-scale studies [70,71]. Along this line, the development of a BLEACH&STAIN multiplex IF pre-trained deep learning model (analyzed above; see Table 1) facilitates the quantification of multiple immune-associated markers with clinical impact [32]. In another study by the same group, an AI-assisted image analysis of 1530 cases with breast carcinoma of no special type led to the discovery of a five-biomarker [PR, ER, androgen receptor (AR), GATA3, PD-L1] prognostic signature [72]. In addition, Mi and co-authors employed digital pathology to capture the intratumoral heterogeneity of the immune landscape in triple-negative breast cancer patients assessing the status of CD3, CD4, CD8, CD20, and FOXP3 [73]. Furthermore, Blom et al. and Carstens et al. reported their robust multiplex assays to investigate the prostate cancer and pancreatic cancer TMEs, respectively [73,74]. Importantly, Carstens et al. demonstrated the importance of studying TILs in the vicinity of cancer cells as they are associated with improved survival in patients with pancreatic adenocarcinoma, demonstrating the clinical value of spatial analysis [73]. This point is in line with the results obtained from Wang et al., showing the predictive impact of T cells in direct contact with cancer cells in TNBC patients (previously analyzed; see Table 1) [31]. Notably, a recent meta-analysis investigating the accuracy of various modalities in predicting response to PD-1/PD-L1 inhibitors found that multiplex IHC/IF exhibited a higher accuracy than PD-L1 IHC, TMB, and gene expression profiling [75]. However, several steps are needed for the implementation of such technologies into routine practice. These include the standardization and validation of each assay (along with its preanalytical, analytical, and postanalytical steps), quality assurance, pathologists’ training, cost-effectiveness analyses, and conducting several reproducibility studies among centers and well-designed clinical trials [11,12].

## 6. Conclusions

Double–multiplex immunostaining helps us capture the complexity of the TME in intact tissues, allowing spatial analysis. An assessment of more than one marker on the same tissue provides functional insight into infiltrating immune cells and unravels the interactions between different components of the TME. Moreover, this approach opens the door for the development of novel biomarkers with improved clinical value. To sum up, accumulating evidence supports the prognostic and predictive impact of different subpopulations of CD8(+) T cells, including proliferating CD8(+)TCF1(+) T cells, as well as subpopulations of CD4(+) T cells and TAMs in invasive breast carcinoma. To this end, additional studies are required that implement double–multiplex immunostainings to assess the role of various immune subpopulations, including resident memory T cells. Importantly, the clinical impact of the tumor immune microenvironment is different among breast cancer subtypes. Additionally, the implementation of digital pathology has revealed the clinical impact of assessing immune cell topography, including the analysis of immune–cancer cell interactions. Therefore, future studies need to take into consideration the spatial distribution of immune cells. Furthermore, the introduction of AI in the assessment of multiplex IF has recently developed immune signatures with prognostic value.

Overall, with the advent of immunotherapy, the staining of more than one immune-related marker on the same section could favor designing individualized immune checkpoint immunotherapies for patients with cancer. It is expected that the employment of digital pathology and the introduction of AI in routine practice will help us maximize our perception of the TME. We expect promising results in the future that will be translated into clinical practice and improve patient care.

## Figures and Tables

**Table 1:** List of immune-associated markers with potential clinical value in invasive breast carcinoma.

Markers	Invasive Breast Carcinoma—Patient Profile	Clinical Outcome	Ref.
CD8(+)/FOXP3(+) ratio	*n* = 110 TNBC patients received neoadjuvant chemotherapy	High CD8(+) TIL and high CD8(+)/FOXP3(+) ratio are associated with improved RFS and BCSS.	[19]
CD4(+)FOXP3(+); CD8(+)FOXP3(+); CD4(+)PD-L1(+); CD8(+)PD-L1(+); CD8(+)PD-1(+)	*n* = 86 patients with invasive breast ductal carcinoma (including *n* = 22 with TNBC)	Low CD4(+)FOXP3(+), CD8(+)FOXP3(+), CD4(+)/PD-1(+), CD8(+)/PD-1(+), and CD8(+)PD-L1(+) levels in patients with TNBC are associated with higher recurrence rate.	[20]
FOXP3(+)CD25(+)	*n* = 43 TNBC patients received neoadjuvant chemotherapy	High FOXP3(+)CD25(+) as well as high PD-L1 (TCs, TILs, CPS) are positively associated with favorable OS.	[21]
CD8(+)PD-1(+)	*n* = 269 TNBC treatment naïve patients	CD8(+)PD-1(+) infiltrates were associated with improved survival, but CD8(−)PD-1(+) infiltrates were not. CD8(+)PD-1(+) independent prognostic marker for improved DFS.	[22]
CD4(+), CD8(+), PD-1(+), TIM3(+), and Cytokeratins	*n* = 50 matched pre-neoadjuvant- and post-neoadjuvant-treated patients	The percentage of CD8(+), CD8(+)PD-1(+), and CD8(+)PD-1(+)/CD8(+) ratio is higher in patients with pCR.	[23]
CD8+Ki-67(+), CD8(+)TCF1(+) and CD163(+)PD-L1(+)	*n* = 791 treatment-naïve patients	High CD8(+), CD8(+)Ki67(+), CD8(+)TCF1(+), PD-L1(+), and CD163(+)PD-L1(+) density are associated with improved DFS in TNBC but not luminal type A. High CD163(+) is associated with worse DFS in luminal type A but not TNBC.	[24]
CD68(+)PCNA(+)	*n* = 110 discovery cohort, *n* = 106 validation cohort	High-CD68(+)PCNA(+) TAMs are associated with increased mortality.	[25]
CD68(+)PCNA(+)	*n* = 102 with neoadjuvant chemotherapy-treated patients	High-CD68(+)PCNA(+) TAMs are associated with decreased RFS.	[26]
CD68(+)CD47(+)	*n* = 282 patients	High CD68(+)CD47(+) is associated with reduced RFS.	[27]
CD68(+)COX2(+) CD163(+)COX2(+)	*n* = 371 patients	High expression of CD68(+)COX2(+) in the tumor stroma and high expression of CD163(+)COX2(+) in the tumor nests predicted worse patient overall survival (OS).	[28]
CD68(+)PD-L1(+)	*n* = 244 TNBC treatment-naïve	High expression of CD68(+)PD-L1(+) stromal cells were associated with improved OS.	[29]
CD8(+)TCF1(+)Ki67(+)	*n* = 279 TNBC enrolled in NeoTRIP randomized controlled trial [30]	High CD8(+)TCF1(+)Ki67(+) density predicts a favorable response to immunotherapy.	[31]
Inflamed PD-L1 + tumor and immune cells	*n* = 587	Inflamed (high CD4, CD8, FOXP3, and DC density) PD-L1 + tumor and immune cells associated with improved OS.	[32]

Abbreviations: BCSS: breast cancer-specific survival; COX2: Cyclooxygenase 2; CPS: combined positive score; DC: dendritic cell; DFS: disease-free survival; FOXP3: Forkhead box protein P3; Ki-67: proliferation marker; OS: overall survival; PCNA: Proliferating Cell Nuclear Antigen; pCR: pathologic complete response; PD-1: programmed cell death 1; PD-L1: Programmed Death Ligand 1; Ref.: reference; RFS: recurrence-free survival; TAMs: tumor-associated macrophages; TCF1: T cell factor 1; TCs: tumor cells; TILs: tumor-infiltrating lymphocytes; TIM3: T cell immunoglobulin and mucin domain-containing protein 3; TNBC: triple-negative breast carcinoma.

## Abbreviations

AI: artificial intelligence; AR: androgen receptor; BCSS: breast cancer-specific survival; CTLA-4: Cytotoxic T-lymphocyte associated protein 4; DFS: disease-free survival; dMMR/MSI-H: mismatch repair deficiency/high levels of microsatellite instability; ER: estrogen receptor; FFPE: formalin-fixed paraffine-embedded tissues; H&E: hematoxylin and eosin; HER2: human epidermal growth factor 2; IBC: invasive breast carcinoma; IF: immunofluorescence; IHC: immunohistochemistry; MHC: major histocompatibility complex; OS: overall survival; PCNA: Proliferating Cell Nuclear Antigen; pCR: pathologic complete response; PD-1: Programmed Death-1; PD-L1: Programmed Death Ligand 1; PR: progesterone receptor; RFS: recurrence-free survival TAMs: tumor-associated macrophages; TCF-1: T cell factor 1 TILs: tumor-infiltrating lymphocytes; TIM-3: T-cell immunoglobulin and mucin domain-containing-3; TME: tumor microenvironment; TMB: tumor mutational burden; TNBC: triple-negative breast cancer; TNM: tumor node metastasis; TSA: tyramide signal amplification.
